# Supplementary material for: Optimizing the robustness of electrical power systems against cascading failures
Source: Sci Rep. 2016 Jun 21;6:27625. doi: 10.1038/srep27625 (PMC4914930; doi:10.1038/srep27625)
Supplement: Supplementary Information [file srep27625-s1.pdf]

SUPPLEMENTARY FILE

Optimizing the robustness of electrical power systems against cascading failures

Yingrui Zhang, Osman Yağan
Department of ECE, Carnegie Mellon University, Pittsburgh, 15213, USA
yingruiz@andrew.cmu.edu oyagan@ece.cmu.edu

A. A proof of Claim 1

Let $x^\star$ denote the smallest solution of $x \geq g(x)$ and $x^{\star\star}$ denote the smallest solution of (23). Since $x \geq g(x)$ automatically gives (23), we always have $x^{\star\star} \leq x^\star$. Here, our goal is to show that

$$\mathbb{P}[S > x^\star] = \mathbb{P}[S > x^{\star\star}] \tag{S.1}$$

If $x^\star$ is also the smallest solution of (23), then the claim follows immediately by virtue of the fact that $x^\star = x^{\star\star}$. Now, assume that there is a solution $x^{\star\star} < x^\star$ of (23). Then it must hold that $x^{\star\star} < g(x^{\star\star})$. From (23), this yields

$$1 = \mathbb{P}[S > g(x^{\star\star}) \mid S > x^{\star\star}] = \frac{\mathbb{P}[S > g(x^{\star\star}), S > x^{\star\star}]}{\mathbb{P}[S > x^{\star\star}]} = \frac{\mathbb{P}[S > g(x^{\star\star})]}{\mathbb{P}[S > x^{\star\star}]}$$

Thus, we have

$$\mathbb{P}[S > x^{\star\star}] = \mathbb{P}[S > g(x^{\star\star})]. \tag{S.2}$$

Key to the proof of Claim 1 is the observation that the function $g(x)$ given at (22) is monotone decreasing in $\mathbb{P}[S > x]$. Put differently, (S.2) implies that $g(x^{\star\star}) = g(g(x^{\star\star}))$, meaning that $x = g(x^{\star\star})$ is a solution of $x \geq g(x)$. Since $x^\star$ is defined to be the smallest of all such solutions, this gives

$$x^\star \leq g(x^{\star\star}). \tag{S.3}$$

On the other hand, the continuity assumption on the distribution of S implies the continuity of $g(x)$. Hence, we have $x^\star = g(x^\star)$. Recalling also that $g(x)$ is monotone increasing in x and that $x^{\star\star} < x^\star$, we get

$$g(x^{\star\star}) \leq g(x^\star) = x^\star. \tag{S.4}$$

Combining (S.3) and (S.4), we conclude that

$$x^\star = g(x^{\star\star}). \tag{S.5}$$

Graphically, this means that the curve $y = g(x)$ is constant (and above the line $y = x$) on the range $[x^{\star\star}, x^\star]$ and it intersects with the line $y = x$ at $x^\star$ (see figure 1 for an illustration).

Combining (S.2) and (S.5), we get

$$\mathbb{P}[S > x^{\star\star}] = \mathbb{P}[S > g(x^{\star\star})] = \mathbb{P}[S > x^\star]$$

which establishes the claim (S.1). ■

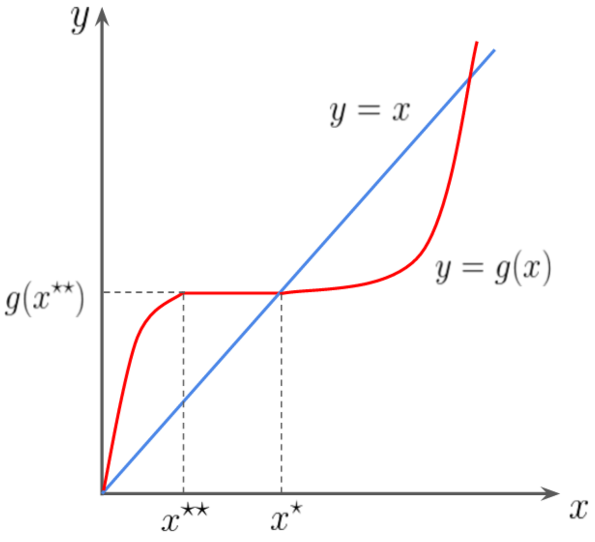

Figure 1. ***Illustration of function relations in Claim 1.*** *We define $x^\star$ as the first point where curves $y = x$ and $y = g(x)$ intersect. We show that if there exists $x^{\star\star} < x^\star$ satisfying (23), then $g(x)$ must be constant over $x^{\star\star} \leq x \leq x^\star$, yielding (S.5).*

B. A necessary condition for abrupt rupture

Recall the condition (30) for an abrupt rupture to take place, namely the need for $h(x)$ to be maximized at $x = S_{\min}$. To explore this issue further, we now compute the derivative of $h(x)$. We have

$$\begin{aligned}
&\frac{d}{dx}(\mathbb{P}[S > x](x + \mathbb{E}[L \mid S > x])) \\
&= \frac{d}{dx}\left(x\mathbb{P}[S > x] + \mathbb{E}\left[L \cdot \mathbf{1}_{[S>x]}\right]\right) \\
&= \mathbb{P}[S > x] + x(-p_S(x)) + \frac{d}{dx}\left(\int_{-\infty}^{\infty}\int_{-\infty}^{\infty} \ell \cdot \mathbf{1}_{[s>x]} p_{LS}(\ell, s) ds d\ell\right) \\
&= \mathbb{P}[S > x] - xp_S(x) + \frac{d}{dx}\left(\int_{-\infty}^{\infty}\int_{x}^{\infty} \ell \cdot p_{LS}(\ell, s) ds d\ell\right) \\
&= \mathbb{P}[S > x] - xp_S(x) + \frac{d}{dx}\left(\int_{x}^{\infty}\int_{-\infty}^{\infty} \ell \cdot p_{LS}(\ell, s) d\ell ds\right) \\
&= \mathbb{P}[S > x] - xp_S(x) - \int_{-\infty}^{\infty} \ell \cdot p_{LS}(\ell, x) d\ell \\
&= \mathbb{P}[S > x] - xp_S(x) - \mathbb{E}\left[L \mid S = x\right] p_S(x). \qquad \text{(S.6)}
\end{aligned}$$

For $x < S_{\min}$, we have $\mathbb{P}[S > x] = 1$ and $\mathbb{E}[L \mid S > x] = \mathbb{E}[L]$, so that $\frac{d}{dx}h(x) = 1$; this is already evident from (29). Then, for an abrupt rupture to take place, (S.6) should turn negative at $x = S_{\min}$; i.e., it must hold that

$$p_S(S_{\min})(S_{\min} + \mathbb{E}\left[L \mid S = S_{\min}\right]) > 1.$$

■
